# Experimental Characterization and Simulation of Thermoplastic Polymer Flow Hesitation in Thin-Wall Injection Molding Using Direct In-Mold Visualization Technique

**DOI:** 10.3390/mi11040428

**Published:** 2020-04-19

**Authors:** Francesco Regi, Patrick Guerrier, Yang Zhang, Guido Tosello

**Affiliations:** Department of Mechanical Engineering, Technical University of Denmark, Building 427A, Produktionstorvet, DK-2800 Kgs Lyngby, Denmark

**Keywords:** glass mold, high-speed camera, thin-wall injection molding, hesitation, simulation, filling

## Abstract

A special mold provided with a glass window was used in order to directly evaluate the flow progression during the filling phase of the injection molding process in a thin-wall cavity and to validate the simulation of the process with particular focus on the hesitation effect. The flow of the polymer was recorded at 500 frames per second using a high-speed camera (HSC). Two unfilled thermoplastic polymers, acrylonitrile butadiene styrene (ABS), and polypropylene (PP), were used to fill two different 50 mm × 18 mm staircase geometry cavities, which were specifically designed to evaluate the hesitation effect with thicknesses of 1500, 1250, 1000, 750, 500 µm (cavity insert no. 1) and 1500, 1200, 900, 600, 300 µm (cavity insert no. 2). In addition to the video recordings, the simulations were validated using the timings and the data obtained by three pressure sensors and two thermocouples located in the cavity. For each injection cycle recorded on camera the machine data were collected to carefully implement the correct boundary conditions in the simulations. The analysis of the video recordings highlighted that flow progression and hesitation were mainly influenced not only by the thickness, but also by the velocity and the material type. The simulation results were in relatively good agreement with the experiments in terms of flow pattern and progression. Filling times were predicted with an average relative error deviation of 2.5% throughout all the section thicknesses of the cavity. Lower accuracies in terms of both filling times and injection pressure were observed at increasingly thinner sections.

## 1. Introduction

The hesitation effect in the filling phase of the injection molding process is a phenomenon that may occur wherever the melt encounters different thickness sections: the melt polymer tends to fill the thicker sections while hesitating in the thin sections. In the worst case, the material freezes before the filling is complete leading to short shots or hesitation marks [1]. The effect can be reduced during the design phase of the part, i.e., avoiding large thickness variations or placing the gate in an opportune position [2]. In this case, modern simulation software is a powerful tool used to improve the design and reduce the lead time to successful production.

However, the design choices may be limited by the complexity of the part, or be relevant only for a type of material. Furthermore, current simulation software presents limits to its predicting capabilities, particularly for thicknesses in the sub-millimeter down to the micrometer dimensional range, as in the case of thin-wall molding.

Wherever pre-machining expedients fail, post design solutions range from the proper optimization of the parameters, to the use of improved processes to facilitate the filling of the cavity [3].

In recent years, glass inserted molds have received great attention as a direct flow visualization methodology in injection molding and other related manufacturing processes. Using these techniques, it is possible to fully investigate the hesitation during the filling phase, validating the simulation of the filling and characterizing the process parameters.

Although the use of glass molds presents some limitations, mostly related to complex-shaped parts, several authors have chosen a glass-inserted mold coupled with various camera and high- speed image recording setups in order to analyze the flow behavior.

Murata et al. [4] and Yokoi [5] obtained simultaneous visualization from two directions thanks to a setup consisting of a camera on the glass side, projecting light through the backside glass and mirrors. Yang et al. [6] characterized a microinjection molding tool and investigated the flow into micro parts with high injection speed. They used a quartz glass window in combination with a reflection mirror and captured videos at 1000 frames per second with a high-speed camera (HSC).

Nian et al. [7] chose the same setup but used different pigments in the polymer blend, they obtained further information about the flow. Han and Yokoi [8] used a higher speed camera capable of recording at 13,500 frames per second and a microscope in order to analyze the filling of micro-grooves on a glass prism insert.

Layser and Coulter [9], investigated the hesitation of the melt flow in the mold cavity. Their results showed that the flow hesitation is primarily dependent on the material, but also on the melt temperature and packing pressure. Similarly, in the present paper, we observed a strong influence of the material, but also of the velocity and geometry used.

Spares et al. [10] analyzed the thermal field generated during microinjection molding with a thermal imaging camera. Whiteside et al. [11] investigated micro molding with a high-speed camera and sapphire glass insert, which allowed them to study diverse effects in microinjection molding.

Recently, Sorgato et al. [12] and Baruffi et al. [13] integrated a sapphire window and a mirror into a tooling system specifically designed for microinjection molding. Thanks to this new setup, they could evaluate the effect of vacuum venting and molding materials on the replication and definition of micro/nano features respectively.

Flow visualization during injection molding has also been performed with mold designs different from those just described. In other solutions, a transparent insert was directly applied to the mold cavity parallel to the injection direction, which allowed the direct filming of the flow from the side without the use of a reflective mirror.

Yanev et al. [14,15] added a sapphire insert to this mold design and performed a simulation comparison to verify the filling performance of the software. Similarly, Guerrier et al. [16] used a mold with a glass window and an integrated multi-turn induction heating to capture the effect of induction heating with a high-speed camera. They then used the same mold to directly compare simulation and experimental moldings from an injection molding machine [17].

The purpose of the present study was to characterize and simulate the hesitation effect in thin-wall molding cavities characterized by a staircase geometry (i.e., exhibiting different thicknesses along the cavity) using the experimental setup presented by Guerrier et al. [16,17], varying the processing parameters in terms of filling settings as well as the type of material, i.e., amorphous acrylonitrile butadiene styrene (ABS) and semi-crystalline polypropylene (PP).

The paper is structured as follows. In the present section the background and motivation of the research work are given and the relevant literature in the field of direct flow visualization is given. In Section 2 the mold construction, injection molding equipment, part design, material characteristics, process settings, high-speed video are detailed. In Section 3 the results of the injection molding experiments are analyzed. In Section 4 the simulation theoretical background, its set up and the results compared with experiments are presented. Section 5 summarizes the paper and conclusions are given.

## 2. Materials and Methods

### 2.1. Glass Mold and Injection Molding Machine

**Figure 1:**
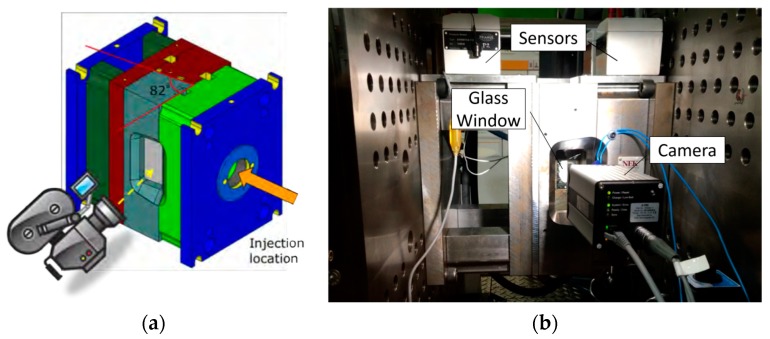
(**a**) Schematic of the mold and of the tilting angle of the glass insert; (**b**) Photograph of the mold setup during an injection molding cycle. It comprises high-speed camera (HSC), glass window and sensors.

A special mold was used in which the injection plane was perpendicular to the opening and closing plane of the glass mold, in order to not hamper the tool opening/closing procedure. The glass was tilted 8°, i.e., 82° with respect to the opening plane, as shown in Figure 1.

This angle was chosen in order to avoid a too tight fit and therefore a possible breakage of the glass when the holding force was applied. The glass window was made of a 55 mm thick borosilicate glass (width 60 mm, height 140 mm), and was capable of withstanding at least 130 MPa of machine pressure during injection, and 50 MPa during packing. In Figure 1b the mold and the position of the camera are depicted.

An X-PRI high-speed camera (AOS Technologies AG, Fislisbach, Switzerland) able to record 1280 × 1024 pixel images at 500 frames per second was used. A spotlight and two light bulbs were installed to increase the brightness of the video. A signaling diode turned on when the filling phase began and turned off at the switch over point. In addition, the mold was provided by three pressure sensors, two thermocouples, two interchangeable inserts, a cooling system, and an ejector system. The mold was installed on an Engel (Schwertberg, Austria) e-motion 110 full-electric injection machine, capable of a maximum clamping force of 1100 kN and equipped with a plasticating screw having a diameter of 25 mm.

### 2.2. Test Parts and Materials

Two inserts with the same geometry were designed to match the constraints imposed by the glass mold geometry and the objectives of the study. The staircase geometry was chosen in order to evaluate the extent of the hesitation and the after-flow in steps of different thickness, particularly in very thin sections where those effects become preponderant. The geometry was comprised of a main channel with a constant thickness of 1.50 mm and several lateral steps with decreasing thicknesses down to the sub-mm range. The two cavity inserts had a very similar design with the only difference being limiting the thickness of the lateral steps.

**Figure 2:**
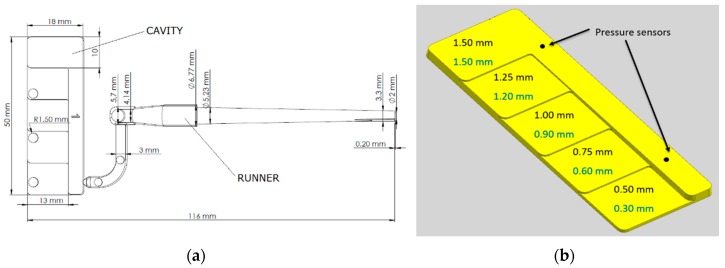
(**a**) Technical drawing of the insert Cavity 1, comprehensive of gate and runner; (**b**) Schematic of the cavity: in black are shown the dimensions of cavity 1, in blue the dimensions of cavity 2. The positions of the two sensors are also shown. Reproduced with permission from [18].

The common dimensions of the inserts are presented in Figure 2a, while in Figure 2b the dimensions of the steps of the first (Cavity 1) and of the second (Cavity 2) insert are shown in black and blue, respectively. Cavity 1 had a thickness of 0.3 mm at the smallest step, it rose by 0.3 mm at each step to a final value of 1.5 mm; Cavity 2, had a thickness of 0.5 mm at the smallest step and it increased by 0.25 mm at each step to a final 1.5 mm. Figure 2b shows also the position of the two pressure sensors in the cavity.

Acrylonitrile butadiene styrene (ABS), a thermoplastic amorphous plastic material and polypropylene (PP), a thermoplastic semi-crystalline opaque polymer were used to characterize the influence of the material on the hesitation.

### 2.3. Experiments

For each material and each cavity the same set of experiments was performed, for a total of 20 runs. The experimental procedure consisted of three complete fillings with a constant injection velocity of 40 mm/s (experiments named “ComF”), 10 mm/s (experiments named “ComM”) and 6 mm/s (experiments named “ComS”) and three short shot experiments (where “-F” stands for fast, “-M” for medium and “-S” for slow injection velocity).

The short shots were obtained using two different methods:

(1) In the maximum pressure (MP) method, the different filling percentages of the cavity were obtained limiting the maximum applied pressure and progressively raising its limit value. MP short shots were therefore produced with variable injection speeds (varying from 4 to 9 mm/s).
(2) In the second method, the desired positions were obtained with consecutive increments of the stroke length at a certain injection velocity. They were addressed as Short Shots (SS). Similar to the complete experiments, SSF runs were with an injection velocity of 40 mm/s, while SSM with 10 mm/s. The velocities were chosen to allow a direct comparison between the complete and the short shot experiments.

To summarize:

- ComF refers to the complete filling experiments with an injection velocity of 40 mm/s;
- ComM refers to the complete filling experiments with an injection velocity of 10 mm/s;
- ComS refers to the complete filling experiments with an injection velocity of 6 mm/s;
- SSF refers to the short shots obtained varying the stroke length with injection velocity of 40 mm/s;
- SSM refers to the short shots obtained varying the stroke length with injection velocity of 10 mm/s;
- MP refers to the short shots achieved limiting the maximum applied pressure.

The data from the experiments ComS were then selected for the simulation analysis and the subsequent validation study.

**Figure 3:**
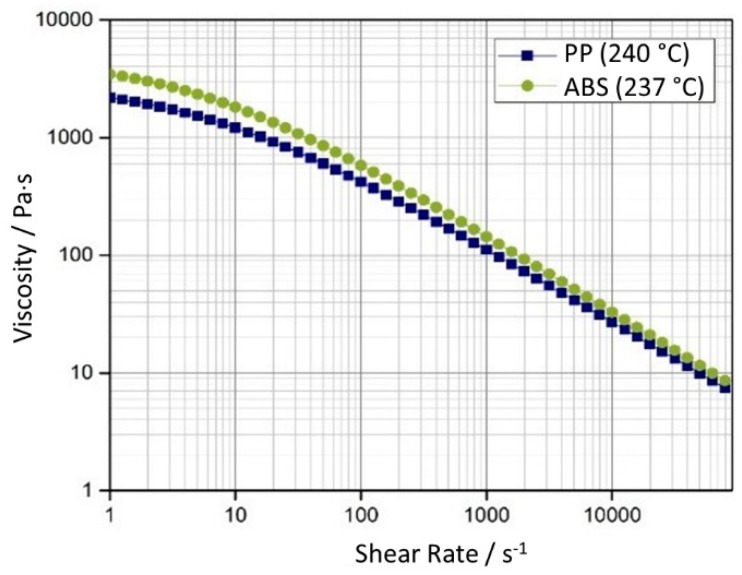
Viscosity of the selected materials at the processing melt temperature.

The rest of the processing parameters were selected based on preliminary optimization experiments as well as following the recommendations of the material suppliers and kept constant throughout the experiments. Cooling time was set to 10 s, with the coolant flowing at 4 L/min at 30 °C. Holding pressure and time were 40 MPa and 4 s respectively for ABS and 40 MPa and 5 s respectively for PP. The mold temperature was set to 40 °C. The melt temperature of ABS was 237 °C, and the melt temperature of PP 240 °C. The viscosity as a function of the shear rate of the two selected materials plotted at their corresponding melt temperatures is presented in Figure 3. For each experiment the actual velocity curves, expressed as ram velocity vs ram position, the machine injection pressure and the sensor data (both temperature and pressure) were recorded.

### 2.4. High Speed Video Analysis

**Figure 4:**
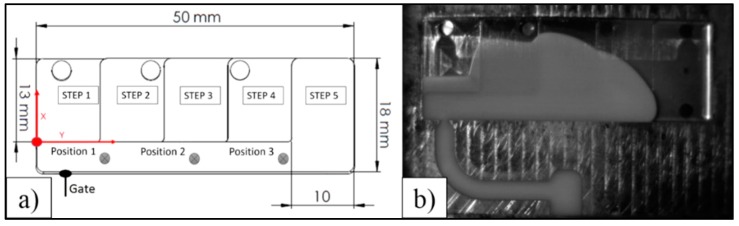
(**a**) Reference system applied in the measurements and the melt positions in the lateral channel considered in the study. These positions are also the final length of the short shots; (**b**) Extracted picture at Position 3 from the video recordings of a complete experiment. Reproduced with permission from [18].

A reference system was set up in order to accurately follow the video recording of the cavity fillings. As presented in Figure 4a, the zero point was at the intersection between the thin step’s external wall and the lateral channel internal wall. The Y-axis was parallel to the channel and the X-axis was perpendicular along the step direction.

Measurements were performed using the open-source Java image processing software ImageJ (National Institutes of Health, Bethesda, ML, USA) [19]. The pixel distances were converted into length units (i.e., mm) with reference to the total length Y of the cavity. The penetration degree X in the side steps was calculated as an average between the maximum penetration and the average penetration on each step. This value gives an estimation of the hesitation for a certain position Y in the lateral channel, which was then used as the reference for the evaluation. Therefore, the results are described by an X distance traveled by the melt flow in the lateral channel at a certain Y length. In the case of the short shots, this was the position where the melt stopped moving (before the retraction caused by the cooling and the corresponding shrinkage), while in the case of the complete experiments, the same position was extracted by the video analysis. Although a total of three positions were chosen, only the results relative to Position 3 (Figure 4a,b), are shown in this paper since these were the most significant. The length of the cavity was measured 12 times in order to evaluate the variability of the measurement method. The resulting standard deviation was 0.3 mm. The related uncertainty was calculated following the guidelines described in [20] and was equal to 0.10 mm.

## 3. Results of Experiments

**Figure 5:**
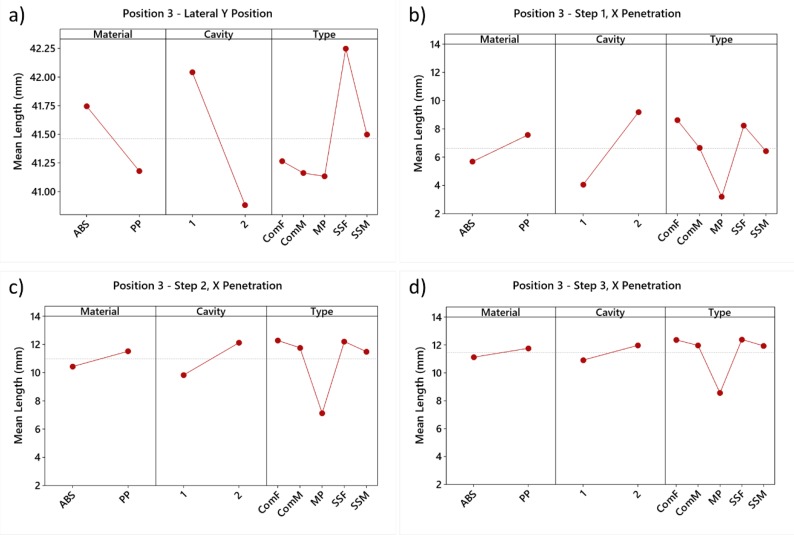
(**a**) Main effects plots for Y length, measure of the accuracy of the position selection, relatively to material, cavity, and type of experiment; (**b**) Main effects plots of the degree of penetration X in Step 1 (thickness = 1.25 mm for cavity 1 and 1.20 mm for cavity 2); (**c**) Main effects plots of the degree of penetration X in Step 2 (thickness = 1.00 mm for cavity 1 and 0.90 mm for cavity 2); (**d**) Main effects plots of the degree of penetration X in Step 3 (thickness = 0.75 mm for cavity 1 and 0.60 mm for cavity 2). Reproduced with permission from [18].

In all the experiments, the length of the melt flow in the lateral channel was chosen to match as accurately as possible the selected position. Therefore, the length Y was a measure of the accuracy of the position selection. Figure 5a shows the main effects plots for Y relative to material, cavity, and type of experiment.

It can be noticed that the traveling distance in the lateral channel is lower in the case of Cavity 2 and the PP experiments than in the case of Cavity 1 and the ABS experiments. A notable difference of the length Y is present between the short shots SS experiments and the experiments performed with a complete filling. When performing a short shot experiment (i.e., SSF and SSM), the flow advances along the cavity (Y-direction, see Figure 5a) more than when a full stroke (i.e., complete filling, ComF and ComM) is injected. On average, the flow front at position 3 obtained with a short shot experiment at high speed (SSF, 40 mm/s) will reach a position in the Y direction 1 mm farther than the corresponding position obtained with a full stroke filling (ComF). At intermediate injection speed (10 mm/s) the flow front of the SSM experiment will reach a position in the Y direction 0.25 mm further than the ComM experiment.

The main effect plots depicted in Figure 5b–d highlight that the hesitation is higher in the ABS experiments, and it is mostly affected by the thickness of the cavity, i.e., higher hesitation in thinner sections (i.e., cavity 2).

The difference between ABS and PP is in agreement with their material properties: PP has, in fact, lower viscosity at the selected melt temperature than ABS (Figure 3). These observations correlate well with flow length studies of similar materials in analogous cavity thickness as presented in [21].

In the case of the MP experiments, the flow was not able to penetrate any of the steps due to the low injection velocity and the limited pressure; therefore, it was not taken into consideration further in.

SS and Complete experiments were in close agreement, but a slight underestimation of the hesitation in short shots SS could be noticed. In Figure 5–d it can be seen that the flow length inside the corresponding section of the cavity (step 1, step 2, step 3 respectively) reached by the melt is shorter for short shot experiments (SSF and SSM) than for complete filling experiments (ComF and ComM). This certainly needs to be considered during the evaluation of filling in molds without glass windows.

It is also worth noticing how the effect of material and cavity diminishes for thinner steps. Similarly, the velocity (i.e., the type of experiment) is more important than the material type for Step 1 (0.5 mm for cavity 1 and 0.3 mm for cavity 2) hesitation, while the opposite is true for thicker steps.

In conclusion, it was possible to make the following observations:

- Thickness variation is the most important factor affecting the hesitation effect;
- The injection velocity and the thickness are not linearly related;
- PP showed less hesitation effect than ABS, as expected from its lower viscosity at the selected melt temperature;
- MP short shot method is not suitable for the characterization of the flow in the cavity;
- Short shots increase the hesitation effect occurring during the filling phase.

## 4. Simulations

The scope of performing the simulations was to validate the ability of currently commercially available software to simulate the hesitation effects. The injection molding simulation software Moldex3D R14 (CoreTech System Co., Ltd., Hsinchu County, Taiwan) was utilized. This study was limited to the experiments carried out with the ABS material since a full characterization of the material data was not available for PP.

### 4.1. Theoretical Background

The material model used in the simulation was based on the Generalized Newtonian Fluid (GNF) model. The three-dimensional non-isothermal flow of the polymer is described by the three main governing equations:

Conservation of Mass:

(1) $$\frac{\partial \rho }{\partial t}+\nabla \cdot \rho u=0$$

Conservation of Momentum:

(2) $$\frac{\partial }{\partial t}\left(\rho u\right)+\nabla \cdot \left(\rho uu-\sigma \right)=\rho g$$

(3) $$\sigma =-pI+\eta \left(\nabla u+\nabla u^{T}\right)$$

Conservation of Energy:

(4) $$\rho C_{p}\left(\frac{\partial T}{\partial t}+u\cdot \nabla T\right)=\nabla \left(k\nabla T\right)+\Phi$$

(5) $$\Phi =\eta {\dot{\gamma }}_{eq}^{2}+\Delta \dot{H}$$

where *u* is the velocity vector, *T* is the temperature, *t* is the time, *p* is the pressure, σ is the total stress tensor, ρ is the density, *k* is the thermal conductivity, $C_{p}$ is the specific heat, ${\dot{\gamma }}_{eq}$ is the equivalent shear rate and $\Delta \dot{H}$ is the heat released during solidification. η is the viscosity as described by the Cross-WLF model. When the infinite shear rate viscosity is negligible the Cross-WLF model reduces to:

(6) $$\eta \left(T,\dot{\gamma },p\right)=\frac{\eta_{0}\left(T,p\right)}{1+{\left(\frac{\eta_{0}\left(T,p\right)\dot{\gamma }}{\tau^{*}}\right)}^{1-n}}$$

where *n* is the Power Law index, $\dot{\gamma }$ is the shear rate and $\tau^{*}$ is the critical shear stress at the transition from Newtonian plateau to shear thinning. $\eta_{0}$ is the zero shear rate viscosity and it is modeled as:

(7) $$\eta_{0}\left(T,p\right)=D_{1}\exp\left(\frac{-A_{1}\left(T-T_{c}\right)}{A_{2}+\left(T-T_{c}\right)}\right)$$

(8) $$T_{C}=D_{2}+D_{3}p$$

where *D*_1_ refers to the viscosity at reference conditions, *A*_1_ and *A*_2_ describe the temperature dependency, *D*_2_ is usually the transition temperature of the polymer, *D*_3_ defines the pressure dependency and *p* the pressure acting on the model.

### 4.2. Simulation Setup

**Figure 6:**
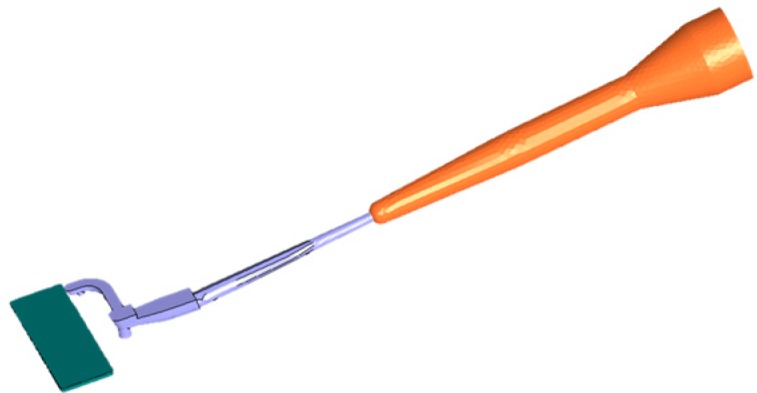
Model of the cavity (green), runner (purple) and screw (modelled as hot runner, indicated in orange).

The simulations were run on an Intel Core i7-5500U 2,4 GHz 4 cores (Intel Corporation, Santa Clara, CA, USA). The scope was to achieve the best possible results in the least possible amount of computational time. The full mold geometry was modeled, including cooling channels, runners, nozzle and barrel geometries, following the procedure described in [16]. Previous studies [16,17,22,23] have shown the importance of considering the full geometry and of implementing the actual injection velocity profile. For this reason, the nozzle and barrel were also modeled as a hot runner (see Figure 6) in order to fully account for the compressible molten material in front of the screw.

**Figure 7:**
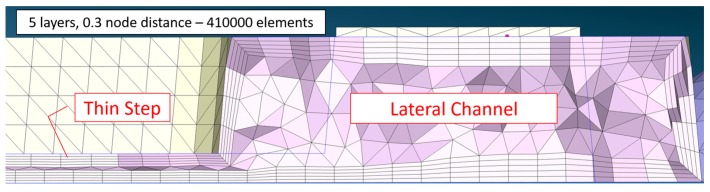
Section of the mesh used in simulations. Five boundary layers and 0.3 node distance were employed in the cavity. Thickness of the lateral channel is 1.50 mm. Thickness of the thin step shown is 0.30 mm.

When meshing the part, a finer mesh was selected for the critical positions in the cavity, whilst a coarser mesh was used for the mold (Table 1). This practice is a well-established methodology to reduce the computational time while maintaining high simulation quality. The mesh consisted of tetra elements and of layers on the boundaries (see Figure 7).

A few tests comprising a significantly higher number of elements (i.e., 4 million elements in total for the part and the runner) were carried out but no significant difference was observed.

The actual process parameters as they were recorded by the injection molding machine and by the sensors were used, i.e., mold temperature and exact switch over point.

The velocity profile was implemented as velocity vs ram position and had two sections: (1) a linear acceleration section needed to increase the speed from 0 up to the set injection speed (e.g., duration of this section was 0.3 s to reach 40 mm/s), followed by (2) a section at a constant value of the set injection speed (e.g., 40 mm/s). Guerrier et al. [17] have in fact shown that considering the screw acceleration in the velocity profile improved the filling time prediction.

### 4.3. Simulation Results

**Figure 8:**
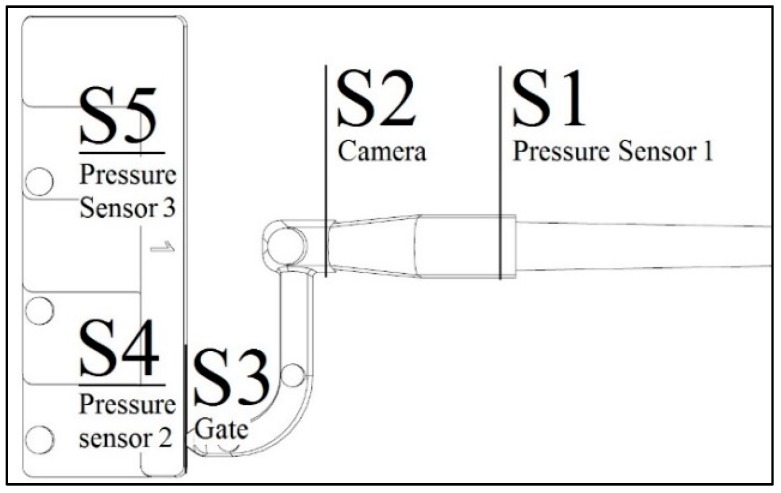
Positions of the sections used to compare experimental and simulated flow. S2 is the entrance in the field of view of the camera. S3 is the gate. S1, S4, and S5 are respectively the first, the second, and the third pressure sensors.

Several sections were selected to characterize the position of the flow front in the cavity (Figure 8). Three sections (namely S1-S4-S5) are in correspondence with the pressure sensors. S2 is the location of the entrance of the flow in the camera’s field of view. S3 is at the gate.

**Figure 9:**
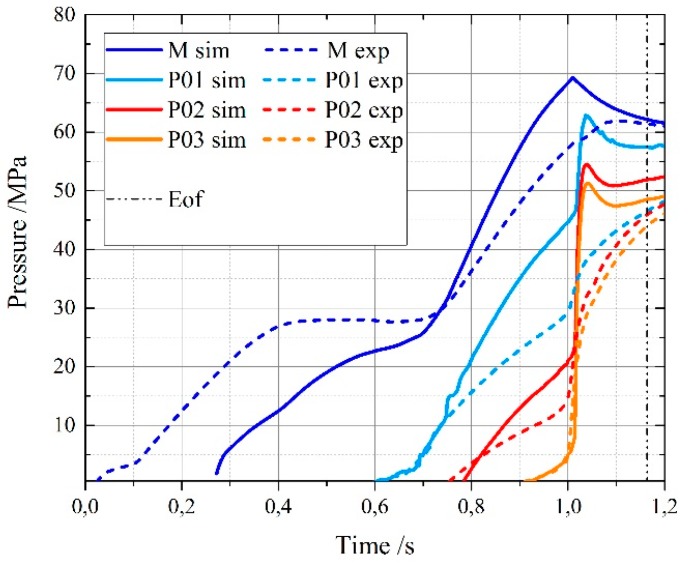
Experimental and simulated pressure curves of ComM-C1. M is the machine pressure. P0X is the sensor pressure. Eof = end of filling.

When comparing experiments and simulations, pressure curves and timings were synchronized to ensure a complete alignment of the switch over point, which was accurately determined, among the different curves. In this way, it was possible to avoid the delay caused by melt compressibility and by the non-return valve. Figure 9 shows how the simulated machine injection pressure at the nozzle (M sim) starts approximately 0.3 s after the real curve (M exp).

Three main factors were compared at the selected sections: flow front pattern (qualitative), the timing and the pressure (quantitative). Figure 9 reports the example relative to the complete filling experiment at medium velocity in cavity 1 (ComM-C1).

The simulated pressures curves present spikes and overestimate the experimental pressure. Several factors may explain this behavior. For example, the viscosity pressure dependency D3 factor was not characterized and considered in the material modeling. As recently investigated by Raha et al. [24] in the case of injection-molded polycarbonate material, an accurate D3 factor characterization can reduce injection pressure underestimation errors down to 10% or less. The enlargement of the section in S1 creates a jetting effect that is not correctly represented in the simulation. Jetting and injection pressure underestimation are related phenomena that are still challenging the currently existing injection molding technology as demonstrated by Tosello et al. [23]. From a structural point of view, the glass window may be subjected to deflections altering the actual pressure in the cavity. Finally, including the modelling of the polymer viscoelastic behavior in the simulation could improve the prediction of flow front prediction and pressure as shown in the case of polystyrene in micro injection molding by Gava et al. [25] and in injection compression molding by Cao et al. [26]. However, a viscoelastic characterization for the materials considered in this study is currently not available and these aspects will need to be investigated in future work in order to evaluate their potential improvement of the simulation accuracy.

**Figure 10:**
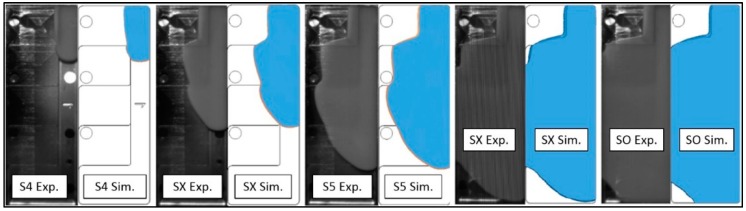
Visual Comparison between experimental and simulated flow front of ComM-C1. The gate position is located at the top right of each image.

The visual analysis was carried out considering melt flow in sections S4, S5, in intermediate positions (SX after S4 and S5 respectively) and at the switch over (SO). The flow front was well predicted in the thicker steps and for the higher velocity, both in pattern and in timing. Figure 10 shows the resulting comparison for experiment ComM-C1 in the thick steps, and the flow front was correctly simulated for the all experiments in the same steps.

**Figure 11:**
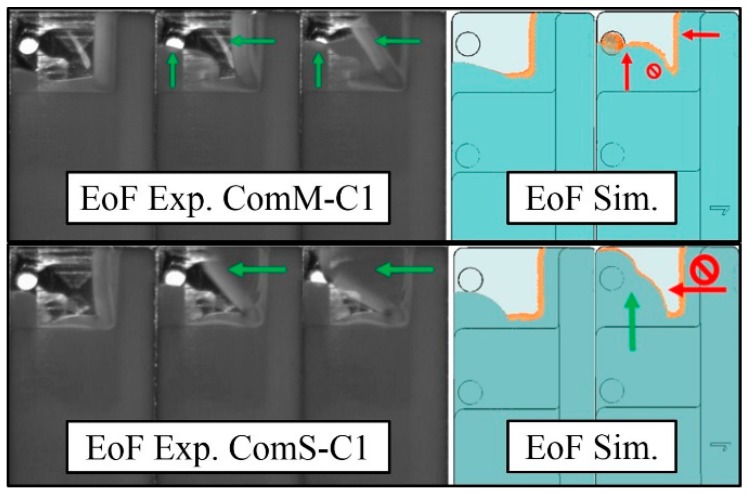
Comparison of the experimental and simulated flow fronts in experiments ComM-C1 and ComS-C1 in the final moments of the filling.

However, in the thinnest step, for the experiments ComM-C1, ComS-C1, and ComS-C2, the software incorrectly predicted a short shot. In the experiments, the flow hesitates in the thin step and proceeds filling the cavity (Figure 11). In the final moments of the filling on the other hand, the pressure is sufficiently high for the melt material to break through the frozen layer and fill the step. In spite of the completed filling, the hesitation may therefore result in evident marks (Figure 11) where the initial frozen layer is located and in weld lines where the flow fronts reconnect during the end of filling (Eof). The software was able to simulate the flow front position but not able to completely reproduce the break-through phenomenon. Instead, it predicted a short shot. Several tests were performed to evaluate the effects of the no-flow temperature and heat transfer coefficient (HTC), however it was not possible to achieve the same optimal results as in Figure 10 when varying these parameters.

**Figure 12:**
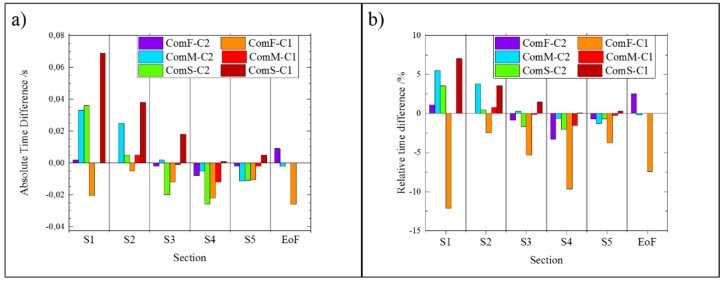
(**a**) Absolute difference between experimental and simulated timings at the selected sections; (**b**) relative difference between experimental and simulated timings at the selected sections.

The timing differences until the end of filling are represented in Figure 12 as the time deviation between the real and simulated fillings at the corresponding positions. The average deviation of the absolute values of the simulated filling times from the experiments was 0.014 s, which corresponds to an average relative deviation of 2.5%. Experiment ComF-C1 was the most challenging for the software to predict with more than 5% of relative difference (higher than the average but still acceptable), whilst for lower velocities there was less than 1% deviation, showing an excellent agreement between simulated and experimental results. These results confirm that modern simulation software can accurately predict the hesitation of the polymer on parts with a section above 500 µm. However, they may not be able to correctly represent the flow for thinner sections, i.e., below 500 µm.

## 5. Conclusions

The present study investigated the hesitation of unfilled thermoplastics through direct flow visualization and simulation. The setup included a transparent insert directly applied to the mold cavity. The cavity consisted of a thin-wall geometry with a series of staircase structures specifically designed to investigate the hesitation effects. The cavity was filled with an amorphous (ABS) and a semi-crystalline (PP) polymer.

The cavity thickness had a strong influence on the hesitation of the plastic melt, whereas the velocity and type of material affected it less. When the thickness decreased down to 300–500 µm, the velocity effect became mainly dependent on the type of thermoplastic. The short shots at consecutive increments of the stroke length overestimated the hesitation effect with respect to complete filling but provided a more accurate representation of the cavity filling.

The flow of ABS was then simulated using Moldex3D R14. The hesitation was well simulated in terms of timings and flow front progress characterization, while some discordances emerged in the pressure levels and in the filling of the thinner step at low injection velocity. Limitations in the accuracy of the predictions were related to challenges in connection with the simulation of jetting, to the presence of deflection of the glass mold insert, and to the effect of the pressure-dependent component of viscosity. Nonetheless, the flow front in the thicker steps was accurately simulated and the timings predicted with a 10% maximum deviation on an average of 2.5%.

## Figures and Tables

**Table 1:** Mesh settings and total amount of elements.

Position	Cavity	Gate	Runner
Layers	5	5	5
Mesh size/mm	0.3	0.3	0.5
Elements/total no.	410,000	255,000	
